# Supplementary material for: A minimal mechanistic model of plant responses to oxygen deficit during waterlogging
Source: Quant Plant Biol. 2025 Jul 21;6:e22. doi: 10.1017/qpb.2025.10016 (PMC12304786; doi:10.1017/qpb.2025.10016)
Supplement: Chen et al. supplementary material [file S2632882825100167sup001.docx]

Supplementary Material accompanying the article

**A minimal mechanistic model of plant responses to oxygen deficit during waterlogging**

**Chen, S.; Boer, H.; Ten Tusscher, K.H.**

**Table S1**. List of parameters in the model

| Parameter type | Parameter | Meaning | Values | Units | References |
| --- | --- | --- | --- | --- | --- |
| Parameters of the plant architecture and the ambient environment | $R_{c}$ | Canopy radius | 0.2 | m | (Wang et al., 2022) |
|  | $Z_{c}$ | Canopy thickness | $2\times{10}^{-4}$ | m | (Tamang et al., 2023) |
|  | $R_{p}$ | Stem radius | 0.01 | m | (Wu et al., 2017) |
|  | $Z_{p}$ | Shoot length | 0.6 | m | (Beegum et al., 2023; Chavarria et al., 2017) |
|  | $R_{r}$ | Root radius | 0.01 | m | (Wu et al., 2017) |
|  | $Z_{r}$ | Rooting depth | $0.3$ (soybean) / 0.2-0.9 | m | (Heatherly & Smith, 2004; Matsuo et al., 2013) |
|  | $R_{rhizo}$ | Radius of rhizosphere | ${2R}_{r}$ | m |  |
|  | $Z_{rhizo}$ | Depth of rhizosphere | $Z_{r}+R_{rhizo}$ | m |  |
|  | $R_{bulk}$ | Radius of bulk soil | ${4R}_{rhizo}$ | m |  |
|  | $Z_{bulk}$ | Depth of bulk soil | $Z_{r}+R_{rhizo}+R_{bulk}$ | m |  |
| Parameters of the plant physiological processes | $A_{max}$ | Maximum rate of oxygen production through photosynthesis per unit leaf area | 30$\cdot{10}^{-6}$ | mol m^-2^ s^-1^ | (Schweiger et al., 2023) |
|  | $\alpha_{AeT}$ | The factor with which maximum cross sectional aerenchyma content increases shoot-root oxygen diffusivity | 2000 |  | (Armstrong & Armstrong, 2014) |
|  | $\alpha_{ROLB}$ | The rate of ROL barrier formation | $1\times{10}^{-5}$ | s^-1^ | Fitted to arrive at a formation process that takes approximately 24 hours (Shiono et al., 2011) |
|  | $m_{g}$ | Glucose molecular weight | 180 | g mol^-1^ |  |
|  | $C_{v}$ | Conversion factor to change from mol/s to atm m^3^ s^-1^ | 10^-5^ RT | Pa mol^-1^ |  |
|  | $\beta_{g}$ | baseline fractional stomatal aperture that can be sustained during waterlogging | 0.1 |  |  |
|  | $\beta_{m}$ | The fraction of baseline carbohydrate-level independent metabolism relative to the total metabolism | 0.2 |  |  |
|  | $D_{plant}$ | Baseline shoot-root oxygen diffusivity without aerenchyma, | $2.15$ $\times{10}^{-8}$ | m^2^ s^-1^ |  |
|  | $D_{shoot}$ | Air-shoot oxygen diffusivity through fully opened stomata | $D_{air}$  $(see below)$ | mol m^-2^ s^-1^ |  |
|  | $h_{O_{2}shoot}$ | Shoot oxygen level leading to half of the aerobic metabolic rate | 0.05 | atm | (Armstrong & Armstrong, 2014) |
|  | $m_{O_{2}shoot}$ | Maximum shoot oxygen consumption rate per unit of carbohydrate through aerobic respiration | $5\cdot{10}^{-8}$ | mol s^-1^ g^-1^ | (Millar et al., 1998) |
|  | $W_{shoot}$ | Shoot dry weight | 10 | g | (Tewari et al., 2007) |
|  | $D_{root}$ | Soil-root oxygen diffusivity under well-drained condition | $D_{soil} (see Eq 9){}$ | mol m^-2^ s^-1^ |  |
|  | $h_{O_{2}root}$ | Root oxygen level leading to half of the aerobic metabolic rate | 0.05 | atm | (Armstrong & Armstrong, 2014) |
|  | $m_{O_{2}root}$ | Maximum root oxygen consumption rate per unit of carbohydrate through aerobic respiration | $5\cdot{10}^{-8}$ | mol s^-1^ g^-1^ | (Millar et al., 1998) |
|  | $p$ | Power of Hill functions for shoot and root aerobic and anaerobic metabolism | 8 |  |  |
|  | $K_{A}$ | Carbohydrate concentration leading to half of the maximum photosynthetic rate | $2\times{10}^{-4}$ | mol g^-1^ | Based on model parametrization |
|  | $n$ | Power of Hill function for dependence of photosynthesis rate on carbon levels | 1 |  |  |
|  | $K_{ATP}$ | Root ATP concentration leading to half of the maximum stomatal aperture | $3.2\times{10}^{-3}$ | mol m^-3^ | Based on model parametrization |
|  | $m$ | Power of the Hill function for stomatal aperture as function of ATP level | 8 |  |  |
|  | $\partial$ | Degradation rate of root ATP | 1 | m^3^s^-1^ |  |
|  | $W_{root}$ | Root dry weight of soybean | 2 | g | (Tewari et al., 2007) |
|  | $h_{rhizo}$ | Rhizosphere oxygen concentration at which oxygen consumption rate is half-maximal | 0.05 | atm |  |
|  | ${\left[ O_{2} \right]^{'}}_{rhizo}$ | Rhizosphere oxygen level that triggers ROL barrier formation | 0.1 | atm |  |
|  | $K_{ROLB}$ | Difference between actual rhizosphere oxygen level and minimum rhizosphere oxygen level triggering ROL barrier formation for which induction is half maximal | 0.1 |  |  |
|  | $q$ | Power of Hill function for ROLB induction | 2 |  |  |
|  | $m_{rhizo}$ | maximum oxygen consumption rate in rhizosphere | $3$ $.15\times{10}^{-3}$ | mol s^-1^ | Based on model parametrization |
|  | $h_{bulk}$ | Bulk soil oxygen concentration at which oxygen consumption rate is half-maximal | 0.05 | atm |  |
|  | $m_{bulk}$ | maximum oxygen consumption rates in bulk soil | $7$ $.64\times{10}^{-5}$ | mol s^-1^ | Based on model parametrization |
|  | $r$ | Power of Hill function for rhizosphere and bulk soil oxygen consumption | 2 |  |  |
|  | $D_{air}$ | oxygen diffusion coefficient when soil pores are completely air filled | $2.02\times{10}^{-5}$ | m^2^ s^-1^ | (Cook & Knight, 2003) |
|  | $D_{water}$ | oxygen diffusion coefficient when soil pores are waterlogged | $2.1\times{10}^{-9}$ | m^2^ s^-1^ | (Cook & Knight, 2003) |
|  | $\theta$ | Fraction of gas filled soil porosity | 0.3/0.65 | m^3^m^-3^ | first value for wet, loamy and second for drier sandy soil |
|  | $f$ | Total soil porosity | 0.5/0.7 | m^3^m^-3^ | first value for wet, loamy and second for drier sandy soil |
|  | $K_{soil}$ | soil water content (height in m) at which soil oxygen diffusion coefficient is $\left( D_{air}+D_{water} \right)/2$ | 0.5 | m |  |
|  | $z$ | Power of Hill function for dependence of soil diffusion on water level | 10 |  |  |
|  | $\left[ O_{2} \right]_{air}$ | Oxygen partial pressure in the air | 0.2 | atm |  |

**Table S2.** Anatomical traits of soybean

| Properties of soybean | Reference value | Value in simulation | References |
| --- | --- | --- | --- |
| Rooting depth | ~0.3 m | 0.3 m | (Heatherly & Smith, 2004; Matsuo et al., 2013) |
| Shoot length | ~0.6 m | 0.6 m | (Vargas Hoyos et al., 2021) |
| Root dry weight | ~3 g | 3 g | (Vargas Hoyos et al., 2021) |
| Aerenchyma content | ~0.2 | 0.2 | (Thomas et al., 2005) |
| ROL barrier | None | 0 | (Ejiri et al., 2021) |

**Figure** **S1**. Comparison between model results and experimental data from Adegoye et al. ([2023](#B1)). The model was parametrized to soybean plant architecture in the respective developmental stage at which the experiments were done (Table S2). (a) soil oxygen concentration, represented by the rhizosphere oxygen concentration in the model, and (b) stomatal aperture, quantified by the ratio between the effective stomatal conductance to the maximum stomatal conductance observed under non-stressed condition.

**Figure** **S2** Dependence of survival time on ROLB absence (a, c, e) versus presence (b, d, f) for $m$ controlling the non-linear dependence of stomatal aperture on ATP set to 2 instead of 8 (a, b), for $n$ controlling the non-linear dependence of aerobic and anaerobic plant respiration on oxygen set to 2 instead of 8, and as compensation $K_{A}$ the saturation constant for this dependence halved (c, d) and a combination of these two changes (e, f).

**Figure** **S3** Dependence of survival time on ROLB absence (a, c) versus presence (b, d) for the default model settings (a, b) and model settings where the negative feedback inhibition of glucose on photosynthesis is removed and replaced by a constant rate.

**Figure** **S4**. Dynamics of (a) root aerobic metabolic rate, (b) root ATP level, (c) stomatal aperture, and (d) root oxygen and rhizosphere oxygen levels of plants with rooting depths of 0.3 m, 0.6 m, and 0.8 m in the absence of aerenchyma and ROL barriers after 20 days (480 hours) upon the initiation of waterlogging. In panel (d), the solid lines refer to root oxygen levels, and the dashed lines refer to rhizosphere oxygen levels.

**Figure** **S5**. Dynamics of (a) shoot-root oxygen transport rate, (b) root aerobic metabolic rate, and (c) stomatal aperture of plants with rooting depths of 0.3 m, 0.6 m, and 0.8 m at aerenchyma content level of 0.5 in the presence/absence of ROL barriers after 20 days (480 hours) upon the initiation of waterlogging.

**Figure** **S6**. (a) Survival time of plants with different rooting depths at different canopy areas; the aerenchyma level stays constant at 05; (b) survival time of plants with different aerenchyma content levels at different canopy areas; the rooting depth stays constant at 0.6m.

**Figure** **S7**. (a) Survival time of plants with different rooting depths at different sensitivity of stomatal closure in response to root ATP levels, with higher $K_{p}$ value representing higher sensitivity; the aerenchyma level stays constant at 50%; (b) survival time of plants with different aerenchyma content levels at different sensitivity of stomatal closure in response to root ATP levels; the rooting depth stays constant at 0.6 m.

**Figure** **S8**. (a) Survival time of plants with different rooting depths at different shoot-root diffusivity levels to oxygen (‘ext low/high diff’ denotes extremely low/high diffusivity). The aerenchyma level stays constant at 50%; (b) survival time of plants with different aerenchyma content levels at different shoot-root diffusivity levels to oxygen. The rooting depth stays constant at 0.6 m.

**Figure** **S9**. The survival time (measured in hours) observed during a 20-day waterlogging treatment across different levels of rooting depths and aerenchyma content levels given different ROL barrier thresholds and thereby initiation time. (a) early ROL barrier induction; (b) reference ROL barrier induction; (c) late ROL barrier induction.

**Figure** **S10**. The difference in survival time associated with varying ROL barrier induction time, on the left between reference induction and early induction (reference - early), and on the right between reference induction and late induction (early - late).
